# The Standard Dictionary of Funk & Wagnalls

**Published:** 1895-01

**Authors:** 


### The Standard Dictionary of Funk & Wagnalls

By actual count contains, exclusive of the Appendix, 301,865 vocabulary words and phrases, and the Appendix of Proper Names, Foreign Phrases, etc., contains 47,468 entries, making the total vocabulary of the dictionary 349,333—this after great care has been exercised to exclude all useless words. The immense increase of the vocabulary of the English language appears from the fact that the vocabulary of Webster's International Dictionary is 125,000 and the Century Dictionary is 225,000.

